# Evaluation of the Corrosion Mechanism of Reinforced Concrete with Surface Treatment of Silica Nanoparticles by Electrochemical Noise Analysis

**DOI:** 10.3390/ma19214551

**Published:** 2026-10-28

**Authors:** Ismael Ricardo Hernández-Cabrera, Aldo Emelio Landa-Gómez, Gerardo Fajardo-San-Miguel, Gabriela Karina Pedraza-Basulto, Jorge Agustín Herrera-Castillo, Edgar Mejía-Sánchez, Ricardo Orozco-Cruz, Andres Carmona-Hernández, Ricardo Galván-Martínez

**Affiliations:** 1 Centro de Investigación en Micro y Nanotecnología, Universidad Veracruzana, Calzada Ruiz Cortines 455, Costa Verde, Boca del Río 94294, Veracruz, Mexico; zs22024505@estudiantes.uv.mx; 2 Facultad de Ingeniería Civil, Universidad Veracruzana, Circuito Gonzalo Aguirre Beltrán s/n, Zona Universitaria, Xalapa 91000, Veracruz, Mexico; aldlanda@uv.mx; 3 Facultad de Ingeniería Civil, Universidad Autónoma de Nuevo León, Av. Universidad S/N, Ciudad Universitaria, San Nicolás de los Garza 66455, Nuevo León, Mexico; 4 Facultad de Ingeniería y Arquitectura, Universidad Autónoma del Carmen, Campus III, Av. Central S/N Esquina Mundo Maya. Colonia Mundo Maya, Ciudad del Carmen 24153, Campeche, Mexico; gpedraza@pampano.unacar.mx (G.K.P.-B.); jherrera@pampano.unacar.mx (J.A.H.-C.); 5 Facultad de Ingeniería, Universidad Veracruzana, KM 1.0 Carretera Sumidero Dos Ríos, Ixtaczoquitlán 94452, Veracruz, Mexico; edmejia@uv.mx; 6 Instituto de Ingeniería, Universidad Veracruzana, Calz Juan Pablo II S/N, Fracc. Costa Verde, Boca del Río 94294, Veracruz, Mexico; rorozco@uv.mx (R.O.-C.); andcarmona@uv.mx (A.C.-H.)

**Keywords:** SiO_2_ nanoparticles, surface treatment, steel–concrete, corrosion, electrochemical noise

## Abstract

This research evaluated the electrochemical effects of using SiO_2_ nanoparticles (NPs) as a surface treatment to reduce porosity in steel–concrete systems exposed to synthetic seawater. Corrosion behavior of steel rebars was investigated using electrochemical noise (EN) measurements, employing several EN analysis methods, including shot noise, Discrete Wavelet transform (DWT), and Hilbert–Huang transform (HHT). SiO_2_ NPs were synthesized from sugarcane bagasse ash and incorporated into concrete systems fabricated with Type 40 composite Portland cement and embedded AISI 1018 carbon steel. The treated systems (designated NP) were subjected to cyclic wetting–drying exposure in synthetic seawater. EN analysis revealed that the reference system (R) exhibited a metastable localized corrosion process. In contrast, the incorporation of NPs modified the microstructure and porosity of the cementitious matrix, thereby modifying the electrochemical response of steel rebars. Shot noise analysis showed corrosion events with lower charge but higher repetition rates, suggesting an increase in the frequency of metastable corrosion processes. Furthermore, DWT and HHT analyses detected periods of increased energy release. This behavior was attributed to a transient stage of a localized process involving partial depassivation, passive film breakdown, and subsequent repassivation. At the end of the exposure period, both systems exhibited partial repassivation, indicating partial recovery of their protective passive conditions.

## 1. Introduction

Corrosion of steel reinforcement is the main deterioration mechanism for reinforced concrete structures (RCSs), often resulting from poor construction practices or prolonged exposure to marine environments [1,2,3]. Once corrosion is initiated, the accumulation of corrosion products generates tensile stresses at the steel–concrete interface, leading to cracking, loss of bond, concrete cover spalling, and a progressive reduction in cross-sectional area [1,2]. This degradation process in RCSs reduces the infrastructure’s load-bearing capacity and service life while increasing inspection, maintenance, and repair costs [3,4]. Consequently, understanding the mechanisms governing the initiation and progression of chloride-induced corrosion remains a subject of scientific interest in the field of reinforced concrete durability [4,5].

The initial protection of embedded steel is attributed to the interaction with the alkaline environment of calcium hydroxide (Ca (OH)_2_), potassium hydroxide (KOH) and sodium hydroxide (NaOH) with pH ≥ 12 of the pore solution and the heat of hydration produced by the hydration of the cement; this condition promotes the formation of a passive film composed of iron oxides and hydroxides, thereby reducing the rate of anodic dissolution [1,6]. However, this passive film is not a uniform, impermeable barrier, as its structure, composition, and ability to reform depend on the electrochemical potential, moisture, oxygen availability, pore solution chemistry [5,6], and the local characteristics of the steel–concrete interface [7].

The steel–concrete interface has a microstructure different from the cementitious matrix [5,7], where interfacial voids, interconnected pores, local accumulation of water, hydration products and oxides coexist [5]. Likewise, local variations in the water/cement ratio (*w*/*c*) and distribution of cement hydration products can lead to areas with different resistivity, oxygen availability and susceptibility to chloride ingress [7]; consequently, the initiation of corrosion may also be influenced by the local properties of the steel and concrete surfaces [5,7].

In marine environments, chloride ions penetrate concrete through the combined action of diffusion, capillary absorption, permeation, and moisture gradient transport [3]. Under cyclic wetting–drying conditions, chloride adsorption during the wetting phase and salt concentration during drying can accelerate chloride accumulation within concrete [3,8]. Some chlorides may become physically adsorbed or chemically bound by the hydrated cement phases, while a free fraction remains in the pore solution, participating in transport processes and the destabilization of the passive film [3,9], as well as influencing the degree of saturation and pore connectivity [8,10]; however, this represents only one part of the reinforcing steel corrosion process.

Moreover, before a persistent anodic region is established, numerous short-lived electrochemical disturbances can occur [11]. Localized defects in the passive film can become activated, resulting in limited iron dissolution and, if conditions are favorable, subsequent repassivation. The repeated occurrence of these processes generates a non-stationary electrochemical response characterized by transients whose amplitude, frequency, and associated charge evolve over time. Consequently, chloride-induced depassivation can be viewed as a sequence of states involving local instability, metastable breakdown, repassivation, the propagation of active sites, and eventually, the establishment of stable localized corrosion [11,12].

One strategy for protecting RCSs is to limit chloride transport while increasing the resistance of the cementitious matrix to ionic ingress through porosity refinement. In this regard, the incorporation of amorphous SiO_2_ NPs has been proposed as an effective approach for modifying the microstructure of cement-based materials [13,14]. Several studies [14,15,16,17] have reported that the incorporation of SiO_2_ nanoparticles improves the compressive strength and durability of concrete. Regarding the mechanisms underlying their beneficial effects, it has been proposed that once incorporated into the concrete matrix, SiO_2_ nanoparticles promote pozzolanic reactions that lead to the formation of a gel-like phase that fills the pores and densifies the cementitious matrix. This phase is mainly associated with the formation of calcium silicate hydrate (C–S–H). This mechanism further refines the pore structure, decreases pore connectivity, and limits the penetration of water and aggressive ions into the cementitious matrix. However, their effectiveness depends on several factors, including dosage, particle size, chemical composition, degree of amorphicity, colloidal stability, and the method of incorporation [16,17].

An alternative approach involves the surface treatment of hardened cementitious materials through immersion in nanoparticle (NP) dispersions [18,19]. NPs can penetrate through accessible pores on the surface, to react with available Ca (OH)_2_ and locally modify the structure of the hardened cementitious material’s hydration products—thereby reducing water absorption and altering chloride transport—though the treatment’s depth and efficiency will depend on the size, degree of agglomeration, and dispersion of the NPs, as well as the connectivity of the pore network [20].

Consequently, the production of NPs from agro-industrial waste such as sugarcane bagasse ash (SCBA) has been studied to improve the performance of cementitious materials with circular economy principles. Owing to its high silica content [21], SCBA can serve as a suitable precursor for the synthesis of amorphous SiO_2_ nanoparticles via the sol–gel method [21,22].

The incorporation of a 0.1% NP suspension has been shown to increase electrical resistivity, reduce porosity, and restrict the transport of ionic species within the hardened cementitious matrix [23,24]. However, these effects do not directly reveal the electrochemical evolution of RCSs. Conventional electrochemical techniques, including half-cell potential measurements, polarization resistance (Rp), and electrochemical impedance spectroscopy (EIS), provide valuable information regarding the average corrosion state of steel embedded in concrete [19,25,26]. Nevertheless, the imposition of an external disturbance (such as Rp and EIS) may limit the detection of short-lived rupture and repassivation events that occur during the initiation of localized corrosion, when active sites can nucleate and disappear stochastically.

Electrochemical noise (EN) is a non-intrusive electrochemical technique that records spontaneous potential and current fluctuations generated by the system’s electrochemical activity without the application of an external perturbation [27]. These fluctuations may contain information related to corrosion processes, making EN a valuable technique for the detection and analysis of the early stages of localized corrosion [27,28].

Several methods have been proposed for the analysis of EN signals. In the time domain, visual inspection of potential (E) and current (I) time series allows transient evaluation, while statistical analysis provides parameters such as the localization index (LI) and noise resistance (Rn) [27,29]. In the frequency domain, noise impedance (Zn) describes the spectral relationship between potential and current signals and requires consideration of signal stationarity, spectral resolution, and concrete ohmic resistance [28,30]. Shot noise analysis represents the electrochemical signal as the superposition of corrosion events based on the power spectral density of E (PSD_E_) and of I (PSD_I_), where B is the Stern–Geary coefficient and A the exposed working area, allowing for the identification of a characteristic charge (q) and the characteristic frequency (fn) associated with corrosion activity [31,32,33,34,35].

In the time–frequency domain, DWT decomposes the EN signals into detail coefficients (called crystals), where D1-D3 are commonly associated with metastable pitting, D4-D6 with localized corrosion, and D7-D8 with diffusion-controlled or uniform corrosion processes [33,36,37]. HHT is suitable for the study of nonlinear and non-stationary signals, by employing the Empirical Mode Decomposition (EMD) to extract intrinsic mode functions (IMFs) [38] and represent the time–frequency evolution of signal energy. Low-frequency energy accumulation has been associated with uniform and diffusion-controlled processes, whereas medium- and high-frequency components are related to localized corrosion phenomena [36,37,39]. However, studies applying HHT to corrosion monitoring in RCSs remain limited. Zhao et al. [40] evaluated the corrosion mechanism of steel embedded in mortar by EN and determined that steel exhibited repetitive processes of passive layer breakdown and repassivation. Permeh et al. [41] observed the pitting corrosion process in post-tensioned bridges by evaluating the effect of adding fly ash to the cementitious matrix under cathodic protection by EN. Hu et al. [42] analyzed the efficiency and mechanism of impressed current cathodic protection for corroded steel reinforcements in concrete by EN, identifying distinct corrosion processes through time-series analysis, Rn, and wavelet analysis. In the present work, the mechanism of chloride-induced corrosion is evaluated in a steel–concrete (SC) system treated by immersion in a 2 g/L NPs dispersion derived from SCBA. The characterization of NPs is integrated with the evaluation of electrical resistivity and nitrogen (N_2_) physisorption of paste made with composite Portland cement 40 (CPC40). Subsequently, the response of the embedded carbon steel was analyzed during drying–wetting cycles in synthetic seawater by EN, employing methods such as I and E time-series analysis, LI, Rn, Zn, shot noise, Wavelet transform and HHT.

## 2. Materials and Methods

### 2.1. Synthesis and Characterization of SCBA-Based NPs

The sol–gel process was used to obtain amorphous NPs according to the method by Falk et al. [22]. The reagents used were: SCBA collected from a sugar mill in Mahuixtlán, Coatepec, Veracruz, Mexico, ground in a ball mill and sieved to obtain a particle size of 75 μm, hydrochloric acid 1 M (HCl, 37%), sodium hydroxide (NaOH) 1 M, distilled water (H_2_O) and filter paper (unifil C41). Briefly, 40 g of ground SCBA was leached with 400 mL of 1 M HCl at 100 °C for 1 h under constant stirring (600 rpm), followed by filtration and washing with distilled water. The leached SCBA was then treated with 340 mL of 1 M NaOH at 100 °C for 1 h under stirring (500 rpm). After filtration, the resulting sodium silicate solution was titrated with 1 M HCl to pH 7 to induce gel formation. The gel was aged for 24 h, filtered, washed with distilled water, and dried at 100 ± 5 °C for 24 h. Finally, the dried product was ground into fine powder.

The NPs were characterized to identify their morphology, a scanning electron microscopy (SEM) instrument at a magnification of 30,000× and energy-dispersive spectroscopy (EDS) were used to identify the characteristic energy peaks of the elements present. X-ray diffraction (XRD) was also used, applying Cukα radiation to powder samples in the 2θ range of 10° to 60° to identify amorphous phases, and X-ray fluorescence (XRF) was employed to determine the chemical composition of the materials in terms of oxides. Particle size distribution was determined via dynamic light scattering (DLS) using samples diluted in propanol and the Fourier transform infrared spectroscopy (FTIR) technique to identify the functional groups present within the 400 cm^−1^ to 4000 cm^−1^ range [21,22,43].

### 2.2. Evaluation of Electrical and Physicochemical Resistivity in Paste Treated with NPs

The reactivity of NPs in hardened CPC 40 paste was evaluated. The paste was prepared in a *w*/*c* ratio of 0.45 in accordance with the ASTM C305 specifications. Table 1 presents the chemical composition of the CPC 40 used in this study, similar to type III cement according to ASTM C150. Cylindrical specimens measuring 30 mm in length and 36 mm in diameter were fabricated from the paste. Twenty-four hours after preparation, the specimens were fully immersed in an aqueous NP dispersion (2 g/L) for 72 h, untreated reference specimens (R) were also prepared [23,24]. The R specimens were cured in a 3.5% *w*/*w* Ca (OH)_2_ solution for 28 days at 23 ± 2 °C (ASTM C511), while the treated specimens (NP) were cured with the same solution for 25 days.

Electrical resistivity was determined by EIS using a potentiostat–galvanostat, following ASTM G106 and the methodology reported in [44]. A three-electrode configuration was employed, consisting of two copper plates (40 × 45 mm) as the working (WE) and auxiliary (AE) electrodes and an Ag/AgCl/KCl_sat_ electrode as the reference electrode (RE).

The measurement parameters used were a frequency range of 1 Hz to 100 kHz with 10 points per decade and a voltage amplitude of 10 mV with respect to corrosion potential (E_corr_). This test was performed on R and NP cylindrical paste samples at 3, 7, 14, 21 and 28 days for each batch of three samples. The electrical resistivity (ρ) of the paste samples was calculated using the following equation:

(1) $$\rho =\frac{\mathrm{RA}}{l}$$

where *l* is the length (3 cm) and *A* is the cross-sectional area (10.18 cm^2^) of the cylindrical paste specimen. *R* represents the paste resistance obtained by fitting the EIS data to the equivalent circuit model [44].

Surface area, average diameter, and total pore volume were determined via N_2_ physisorption. This test was performed using a surface area analyzer based on the Brunauer–Emmett–Teller (BET) method. Cubic samples measuring 5 mm on each side and weighing 0.5 g were taken, extending from the surface toward the interior. A degassing treatment was carried out at 120 °C for 24 h, after which the samples were analyzed under vacuum at −196 °C.

### 2.3. Preparation of Untreated and Surface-Treated Steel–Concrete (SC) Specimens

The dimensions and geometry of the SC systems were fabricated following a procedure similar to that reported by Landa-Gomez et al. [19]. The following materials were used: CPC 40 with strength class 40 (ASTM C150); andesite coarse and fine aggregates with nominal maximum sizes of 12 mm and 4.75 mm, respectively (ASTM C33/C33M); AISI 1018 corrugated steel (ASTM A615/615 M) bars 9.5 mm in diameter and 120 mm in length, with an exposed length of 50 mm (14.9 cm^2^); a 12-gauge THW-type electrical conductor; epoxy coating to delimit the exposure area of steel; 12.5 mm diameter chlorinated polyvinyl chloride (CPVC) pipes; epoxy plasticine to encapsulate and insulate the copper wire-to-steel bar connection; elastomeric coating to delimit the concrete exposure area; a *w*/*c* ratio of 0.45; and a 28-day compressive strength of 38 MPa. Table 2 shows the mix design used, which met the specifications of the ASTM C94/C94M standard. Figure 1 shows the schematic representation of the SC systems.

Surface treatment of the SC systems was performed 24 h after fabrication; it was applied via total immersion in an aqueous solution containing SiO_2_ nanoparticles for 72 h. The nanoparticles were dispersed in distilled water at a concentration of 2 g/L using an ultrasonic bath for 5 min. The R system was cured in a 3.5% Ca (OH)_2_ solution for 28 days at 23 ± 2 °C (ASTM C511), while the treated system (NP) was cured in the same solution for 25 days. Following the treatment and curing periods, the SC systems were subjected to drying (3 days) and wetting (4 days) cycles in synthetic seawater (ASTM D1141).

### 2.4. Electrochemical Noise Measurements

EN measurements were conducted at room temperature using a three-electrode cell configuration in accordance with the ASTM G199 standard. Two nominally identical AISI 1018 steel bars embedded in the SC systems were employed, denoted by WE1 and WE2, along with an Ag/AgCl/KClsat RE, while synthetic seawater served as the electrolyte (Figure 2). EN measurements were recorded at a sampling frequency of 1 Hz in blocks of 1024 data points using a potentiostat/ZRA (zero-resistance ammeter) after completion of the wetting period. Measurements were conducted on days 3 and 7 and subsequently every 7 days.

For time-domain analysis, EN signals were examined by visual inspection of the I and E time series, as well as by calculating statistical parameters. LI was determined as the ratio of the current standard deviation to its root mean square value, whereas Rn was calculated from the ratio of the standard deviations of potential and current noise. For frequency-domain analysis, noise impedance (Zn) was calculated from the ratio of the power spectral density (PSD) of the potential and current signals. Prior to transformation into the frequency domain, a Hann window was applied, and the Fast Fourier Transform (FFT) was performed. For time–frequency-domain analysis, energy distribution plots (EDPs) were generated using a decomposition level of j = 8 and the fourth-order Daubechies orthogonal wavelet function (Db4) applied to the EN signals. In addition, the Hilbert–Huang Transform (HHT) was employed to extract intrinsic mode functions (IMFs) through Empirical Mode Decomposition (EMD) and to obtain the instantaneous frequency distribution using the Hilbert spectrum. The FFT, wavelet transform, EMD, and Hilbert transform procedures were implemented using the corresponding MATLAB toolboxes (2023b version).

## 3. Results and Discussion

### 3.1. Characterization of SiO_2_ NPs

Figure 3 shows the micrograph of the NPs obtained from SCBA. Particles of varying sizes with rounded and irregular edges were identified; smaller particles were observed to be adhering to larger ones. During the synthesis process, NP nucleation generated capillary forces and siloxane/silanol bonds that promoted the coalescence of primary particles (smaller ones) into secondary conglomerates (agglomerates). Consequently, the agglomeration of NPs synthesized from agro-industrial waste may be attributed to the presence of residual salts and variations in impurity content, which alter colloidal stability [45,46].

EDS analysis (Figure 4) revealed higher percentages of O and Si, as well as residual traces of other elements in lower proportions—such as Al, Fe, K, and Na—attributed to harvesting, storage, and fertilization processes, as well as the synthesis itself [47].

The NP size was determined via DLS, as shown in Figure 5. Two predominant ranges were identified: the first was between 70 and 100 nm (peaking at 80 nm) and the second between 200 and 450 nm (peaking at 300 nm). This aligns with SEM observations, indicating that the resulting NPs consist not only of “dispersed primary particles” (particles smaller than 100 nm) but also of secondary aggregates (larger than 100 nm) [48,49,50].

Figure 6 shows the diffractogram of the NPs. A broad halo characteristic of predominantly amorphous SiO_2_ was identified in the 22–23° (2θ) region [51]. In addition, two sharp peaks were observed at 31.7° and 45.4° (2θ), attributed to halite (NaCl); this likely resulted from the chemical synthesis process, in which silica extraction from SCBA typically involves first forming sodium silicate via alkaline treatment (NaOH) and subsequently inducing gelation/precipitation with an acid (e.g., HCl), leading to the formation of soluble salts such as NaCl that remained as crystalline residues despite washing steps or due to recrystallization during drying [52,53].

Figure 7 shows the infrared spectrum of the NPs obtained by FTIR. Characteristic groups of SiO_2_ xerogels were identified [54]. The first was located at 1047 cm^−1^, attributed to the asymmetric stretching vibration of Si-O-Si; the second peak was at 792 cm^−1^, corresponding to the symmetric stretching vibration of the Si-O-Si bond [54,55]; and a third peak at 418 cm^−1^ was related to bending vibrations of the Si-O-Si bond [55]. This is because the solubilization of sodium silicate using HCl promoted the formation of silanol groups, whereas siloxane groups formed through condensation [56]. Furthermore, the presence of water molecules adsorbed onto the silica surface was attributed to the O–H stretching vibration of silanol groups (Si–OH)—observable in the 3000–3700 cm^−1^ range [54]—such that the peak identified at 3412 cm^−1^ is associated with hydroxyl groups (O-H) [51], while the peak at 1630 cm^−1^ is associated with the H_2_O bending vibration of the silanol group (Si-OH) [55,57]. The absence of absorption bands between 1700 and 2800 cm^−1^ associated with organic residues and certain impurity-related species suggests a reduction in these components [58]. However, this FTIR observation cannot be considered evidence of the complete removal of organic compounds, metals, or other impurities.

Table 3 presents the results regarding the chemical composition of the obtained nanoparticles (NPs). This quantitative chemical analysis via XRF reveals that the NPs synthesized from SCBA using the sol–gel process achieved a purity of 90.6%; traces of other oxides [45,47,59] and chlorine residues resulting from the synthesis process were also observed. This residual chlorine content is considered to make a negligible contribution to the chloride-induced corrosion process. The estimated chloride concentration in the surface treatment solution is ~69.44 mg/L; therefore, the amount of chloride that could potentially diffuse through the concrete and reach the reinforcing steel is expected to be very limited.

### 3.2. Textural and Electrical Resistivity Properties of NP-Treated Paste

Figure 8 shows the results obtained for electrical resistivity (ρ) as a function of NP dosage and curing time in the hardened paste samples. In general, the evaluation revealed a pattern of increasing resistivity consistent with cement hydration and the formation of products such as C-S-H; thus, the increase in ρ over time suggests microstructural densification and a reduction in porosity within the paste [60,61,62].

Differences were observed between the R paste and the treated paste, implying an additional effect induced by the NPs. After 72 h (3 days) of treatment, the NP paste exhibited 33% higher resistivity compared to the R paste; this suggests that the NPs contributed by creating nucleation sites and acting as a reactive source of amorphous silica capable of consuming portlandite and promoting additional C-S-H formation [63,64]. Subsequently, at 7 days, similar values were observed for both paste types. At 14 days, the NP paste continued to show higher resistivity, 18% greater than the R paste. By day 21, the treated paste still exhibited a resistivity value 12% higher than the R paste. At 28 days, no significant differences were observed between the treated paste and the R paste; it is inferred that following NP treatment, the curing process fostered a highly alkaline, stable environment and continuous hydration within the cementitious matrix, thereby reducing the treatment’s relative advantage. These results are consistent with findings reported for NP treatments, which facilitate pore blocking and physicochemical changes associated with the localized formation of denser hydration products [60,65].

Figure 9a shows the pore size distribution for NP and R paste at 3 and 28 days. At 3 days, the R paste exhibited the highest peak of the entire series in the fine-pore region—approximately between 1.5 and 2.5 nm—as well as another region between 4 and 10 nm; this indicates that at an early age, the cementitious matrix retained a significant fraction of pores in the fine- and small-mesopore ranges, as cement hydration was still limited, the C-S-H network was still consolidating, and fine-pore connectivity remained relatively high. In contrast, the NP paste showed a decrease of approximately 45% in pores within the 1.5 to 2.5 nm range while maintaining porosity between 4 and 10 nm, suggesting that the NPs formed additional C-S-H through a pozzolanic reaction with Ca (OH)_2_, thereby reducing porosity in the fine-pore fraction. At 28 days, both the NP and R paste showed a similar reduction in porosity between 1 and 2 nm; however, the NP paste exhibited a porosity reduction of approximately 33% compared to the R paste in the 2 to 10 nm range [66,67,68].

Figure 9b shows the N_2_ adsorption isotherms for the NP and R paste. At 3 days, the R paste exhibited a progressive increase in the amount adsorbed at high relative pressure (*p*/p0 > 0.8), suggesting a higher abundance of mesopores where capillary condensation occurred [66]. In contrast, the NP paste showed reduced adsorption at intermediate pressure, yet adsorption at high pressure was 40% higher than that of the R paste; this implies that pore blockage was still limited at this stage, as the NPs were still integrating into the cementitious matrix—initially blocking micropores while leaving a network of mesopores unobstructed—since the initial effect was likely localized and dependent on NP penetration [20,68].

At 28 days, the R paste exhibited reduced adsorption at intermediate pressure compared to the 3-day sample, suggesting the conventional progression of cement hydration and a reduction in micropores; however, an increase was observed in the high-pressure zone, attributed to the continued presence of mesopores where capillary condensation occurred [69]. Conversely, the NP paste at 28 days showed lower adsorption across the entire relative pressure range, indicating a reduction in mesopore volume and capillary condensation, which is attributed to the additional formation of C-S-H resulting from the interaction between the NPs and the Ca (OH)_2_ present in the cementitious matrix [66,67]. It should be noted that N_2_ physisorption characterizes only the accessible porosity within the micro- to mesopore range, is influenced by the technique’s drying pretreatment, and does not represent the total porosity [68].

Table 4 presents the results obtained from the N_2_ physisorption test. In the NP paste, the surface area decreased by 25% to 28.5% at both 3 and 28 days compared to the reference (R); this is associated with the growth and densification of the additional C-S-H gel formed through the interaction with the NPs during the hydration process [68]. This behavior was also observed regarding pore volume over the same period, which decreased by 5.5% to 20%, indicating that the reduced adsorption was not merely a surface effect but a genuine decrease in the paste’s accessible porosity [20,67]. Furthermore, although 12% and 1% increases in average pore diameter were observed during the evaluated period, they did not indicate structural deterioration of the treated paste; instead, they were attributed to a selective redistribution of porosity. Specifically, micropores were occluded by the formation of additional C-S-H resulting from the interaction between NPs and Ca (OH)_2_, manifesting as an increase in apparent average diameter without increasing the total pore volume—a finding that correlates with the observations in Figure 9 and Figure 10 [66,67].

### 3.3. EN Measurements

#### 3.3.1. Time-Domain Analysis

Figure 10 shows the current and potential time series for the R and NP systems at 3, 35, 91, 119, and 182 days of exposure. On day 3, the R system exhibited steel passivation behavior characterized by passive film breakdown followed by partial repassivation (Figure 10a), accompanied by a decrease in potential from −0.222 to −0.233 V and a current fluctuation amplitude of ~0.8 × 10^−6^ A. In contrast, the NP system (Figure 10b) displayed a stable passivation process on day 3 along with less electronegative potential values compared to the R system. Following the first wet–dry cycle, at day 35, both specimens (Figure 10c,d) showed an increase in the amplitude of potential and current transients, indicating the onset of chloride ingress. At day 91 (Figure 10e), the R system exhibited a new localized passive film breakdown event associated with a potential drop of ~5 mV. Similar abrupt transients have been previously associated with localized corrosion processes in reinforced concrete systems [70]. In contrast, the NP system (Figure 10f) displayed higher amplitudes of current and potential transients, indicating that Cl- ions had likely reached regions adjacent to the steel surface. However, the absence of abrupt events suggests that the nanoparticle treatment may delay the onset of depassivation and localized corrosion processes [26].

At day 119, the R system (Figure 10g) exhibited periodic current transients of ~9 × 10^−8^ A, accompanied by potential transients of ~0.8 mV and a potential decrease of ~5 mV, suggesting activation–repassivation processes. In contrast, the NP system (Figure 10h) showed larger current (~2.8 × 10^−7^ A) and potential (~1 mV) transients, indicative of metastable localized corrosion. These results suggest that although the NPs modified the cementitious microstructure and hindered chloride transport, localized depassivation processes were not completely suppressed [15]. By day 182 (Figure 10i,j), both specimens exhibited similar behavior, characterized by high-frequency, low-amplitude current (~2 × 10^−8^ A) and potential (~0.8 mV) transients, indicating stabilization of the corrosion process.

**Figure 10:**
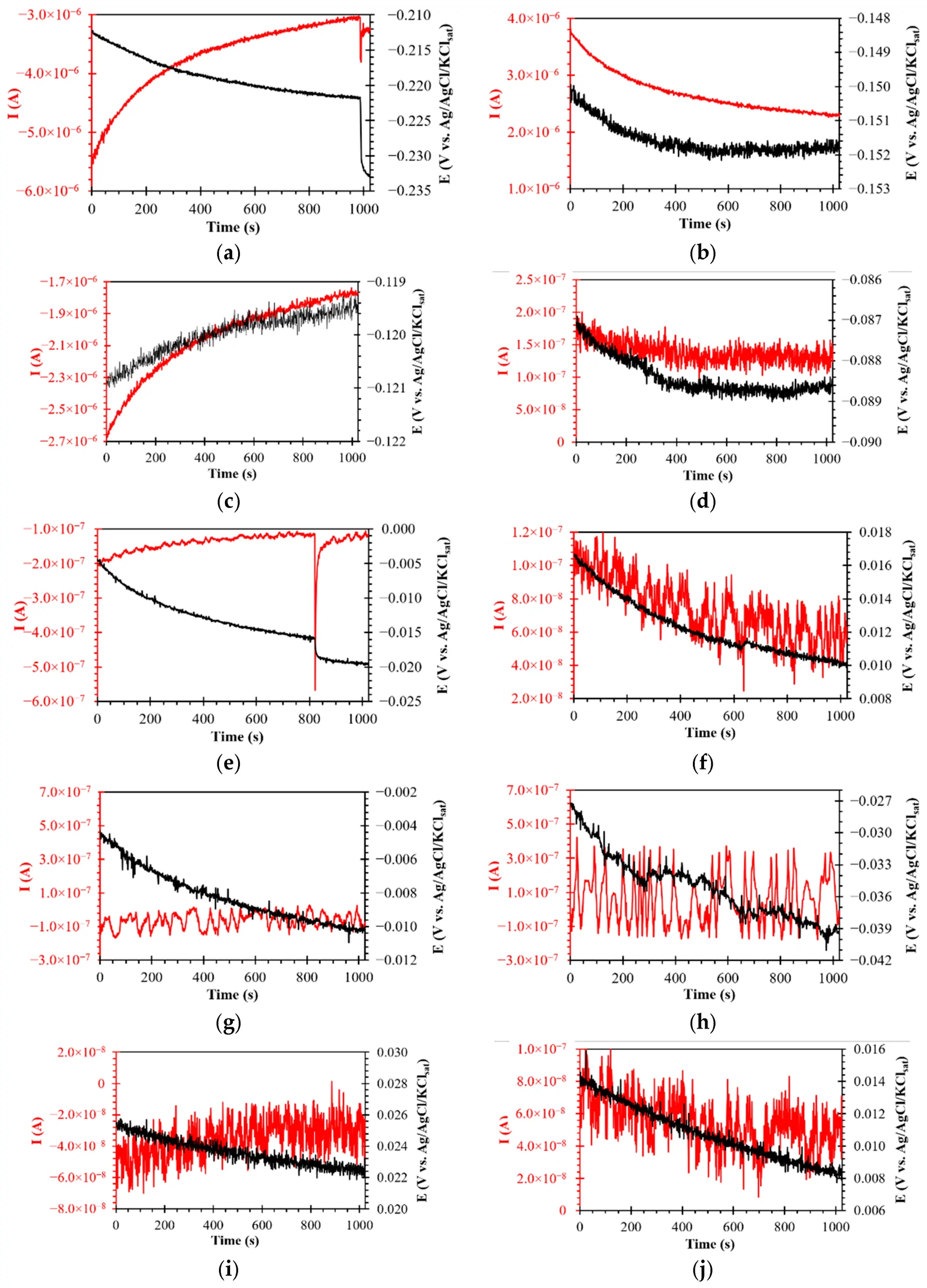
Current and potential time series for R (**a**,**c**,**e**,**g**,**i**) and NP (**b**,**d**,**f**,**h**,**j**) systems at different days of exposure: 3 (**a**,**b**), 35 (**c**,**d**), 91 (**e**,**f**), 119 (**g**,**h**) and 182 (**i**,**j**) days.

Figure 11a shows the LI values for the R and NP systems. Throughout the exposure period, LI remained within the localized corrosion range, likely due to passive film formation and metastable pitting activity. During curing, both systems exhibited stable behavior; however, after the first wetting–drying cycle (day 35), the LI of the R system decreased from 0.6 to 0.4, consistent with chloride ingress, as also suggested by the increased transient amplitudes observed in the EN signals. In contrast, the NP system exhibited a gradual decrease from 0.7 to 0.3 up to day 49 (the third wetting–drying cycle), indicating that the nanoparticle treatment modified the pore network and delayed, although not completely prevented, Cl^−^ penetration.

Figure 11b presents the Rn values obtained for the R and NP systems. For the R system, Rn remained below 50 kΩ*cm^2^ up to day 35, followed by a progressive increase to a maximum of 1250 kΩ*cm^2^ at day 91, coinciding with the passive film activation–repassivation event noted in the time-series data. Thereafter, Rn stabilized at 200 kΩ*cm^2^. The occurrence of this maximum may be associated with the temporary formation of corrosion products that increased resistance to current flow without suppressing localized charge-transfer events. In contrast, the NP system exhibited a gradual increase in Rn values from 50 to 200 kΩ*cm^2^ by day 35, suggesting that the nanoparticle treatment reduced pore connectivity and hindered chloride transport. A subsequent decrease to 75 kΩ*cm^2^ was observed at 49 days, similar to the R system. A maximum of 1000 kΩ*cm^2^ was also observed at 98 days, indicating that the NP-modified matrix limited corrosion-product formation compared to the R system. Thereafter, Rn values remained generally higher than those of the R system, likely due to localized chloride accumulation within partially blocked pores, promoting metastable localized corrosion events [28,70].

#### 3.3.2. Frequency-Domain Analysis

Figure 12 shows the Zn spectra of the NP and R systems. The R system exhibited values tending to increase toward ~10^3^ Ω*cm^2^ between days 3 and 28, driven by the formation of a passive layer on the steel surface, promoted by the highly alkaline pore solution. On day 35, a deviation from this increasing trend was observed, coinciding with the onset of chloride ingress, as also evidenced by the LI values. This behavior suggests the emergence of electrochemically active areas and a partial instability of the passive film. Subsequently, between days 91 and 154, Zn decreased, indicating that the system became increasingly influenced by localized corrosion processes involving dissolution of the passive film. During the final exposure period (154–182 days), Zn values showed recovery, which can be attributed to the accumulation of corrosion products at the steel–concrete interface. These corrosion products likely hindered ionic transport and increased the resistance to current flow, although without fully restoring passivity [28,71].

Figure 12b shows the Zn values for the NP system. Following NP treatment and the curing process, the behavior of the Zn values differed markedly from that observed for the R system, with values remaining close to or above 10^4^ Ω*cm^2^ in the low-frequency range. This suggests that the treatment modified the pore structure and connectivity of the cementitious matrix, thereby reducing the mobility of electroactive species and increasing the resistance of the system [3,15,26]. However, on day 154, the Zn value decreased to ~10^3^ Ω*cm^2^. This variation is attributed to the initiation of recurrent activation–repassivation cycles on the steel surface after the Cl^−^ concentration at the steel–concrete interface reached a critical threshold. By day 182, Zn increased again, likely as a consequence of repassivation processes, similar to the behavior observed for the R system. The NP treatment promoted the formation of a denser cementitious matrix and increased the overall resistance of the system by partially blocking pore pathways. As a result, Cl^−^ transport became more restricted, leading to steel activation at specific sites where critical concentrations of chlorides, oxygen, and moisture were simultaneously attained.

Figure 13 shows the shot noise results, showing the charge and characteristic frequency of corrosion events for the R and NP systems. A constant B value of 26 mV was assumed for steel in the active state and 52 mV for the passive state [19,35]. For both systems, a cluster of data points appears in the lower-right quadrant, corresponding to low-charge, high-frequency events typically associated with slight uniform corrosion or passivation [33]. These events are attributed to the presence and stability of the passive film. As the drying–wetting exposure progressed, the R system exhibited a shift toward the upper-left region of the plot, which is indicative of localized processes characterized by higher charge and lower frequency. This behavior is attributed to Cl^−^ ingress and the consequent localized breakdown of the passive film, resulting in corrosion events with charges as high as ~3 × 10^−6^ C. In contrast, the NP system displayed a less pronounced displacement toward the localized corrosion region, indicating lower susceptibility to passive film breakdown. The maximum charge recorded for this system was ~5 × 10^−7^ C, nearly one order of magnitude lower than that observed for the R system. This behavior suggests that the NP treatment effectively limited Cl^−^ ingress and delayed the development of localized corrosion processes [33,72].

#### 3.3.3. Time–Frequency-Domain Analysis

Figure 14 displays the energy distribution plots (EDPs) obtained from the application of the DWT to EN current signals. In these plots, the EN signal is decomposed into so-called “wavelet crystals” (j), corresponding to detail levels or coefficients—which correspond to detail coefficients representing the relative energy contribution of specific frequency bands within the signal [36,37].

Figure 14a and Figure 14b show the EDPs for the R and NP systems, respectively. Between days 3 and 91, both systems were dominated by diffusional processes and passive film formation, with the highest energy contributions concentrated in crystals j = 7–8. Subsequently, the energy shifted toward intermediate scales, reflecting the increasing contribution of localized and mixed corrosion processes.

For the R system, an energy transition at j = 5–6, accompanied by a reduction in the energy contribution at j = 8, was observed between days 91 and 119. This behavior is attributed to passive film destabilization caused by chloride ingress and redistribution, leading to the onset of localized corrosion activity. In contrast, the NP system exhibited a shift toward crystals j = 4–6 between days 91 and 119 and at j = 5 between days 119 and 154. These transitions are associated with localized depassivation–repassivation events, redistribution of corrosion products, changes in internal saturation, and modifications in ionic transport pathways within the NP-treated cementitious matrix [36].

From days 154 to 182, both systems exhibited a renewed concentration of energy in crystal j = 8, indicating a return to a mass-transport-controlled regime. This behavior may be associated with the accumulation of corrosion products, partial pore blockage (particularly in the NP system), and changes in the internal degree of saturation, which collectively contributed to the stabilization of the electrochemical response after prolonged drying–wetting exposure.

Figure 15 shows the HHT spectra for the R and NP systems derived from the EN current signals. In HHT analysis, characteristic frequencies of corrosion events can be associated with specific frequency ranges. During the early stages (at 3 days), both systems (Figure 15a,b) exhibited energy concentrated in the low-frequency range (10^−3^ to 10^−2^ Hz), indicating long-term processes associated with passive film formation under the highly alkaline curing conditions.

For the R system, the onset of drying–wetting cycles led to a progressive shift in energy toward the intermediate-frequency range (10^−2^ to 10^−1^ Hz), particularly on days 35 (Figure 15c) and 91 (Figure 15e). This behavior is consistent with the pronounced changes in Rn and Zn values, indicating a critical transition from passive stabilization to chloride-induced localized depassivation. The increased energy can be attributed to the local passive film breakdown and subsequent repassivation.

The NP system (Figure 15d,f) exhibited a similar pattern to the R system but showed higher contribution in the mid-frequency range, reflecting the onset of chloride-induced passive film disruption. However, unlike the R system, the response was characterized by a more uniform energy distribution rather than a dominant high-energy event. This behavior suggests that depassivation occurred more gradually and was partially mitigated by the altered transport properties of the NP-modified cementitious matrix.

After 119 days, both systems (Figure 15g,h) exhibited a broader energy distribution within the intermediate-frequency range, indicating a metastable localized corrosion regime characterized by recurrent electrochemical transients. These events are consistent with repeated passive film breakdown, short-lived anodic activation, and subsequent repassivation processes.

At 182 days, both systems (Figure 15i,j) continued to exhibit distributed energy contributions, indicating that a fully passive state was not reestablished. Instead, the systems evolved toward a quasi-stable electrochemical regime in which corrosion activity persisted under the influence of mass-transport limitations, corrosion product accumulation, and the redistribution of anodic and cathodic sites. Consequently, the energy response was concentrated primarily in the low-to-medium frequency range (10^−3^ to 10^−1^ Hz) at relatively low amplitudes, indicating the predominance of slow processes associated with oxygen diffusion, chloride transport, internal moisture fluctuations, and the polarization of the steel–concrete system [33,37].

### 3.4. Corrosion Mechanism in the RCS with and Without SiO_2_ NPs

The electrochemical response obtained in this study indicates that chloride-induced corrosion did not occur as an instantaneous degradation process. Instead, both systems evolved through a sequence of interfacial changes involving passive film stabilization, chloride ingress, localized depassivation and metastable corrosion activity. The main difference between the R and NP systems was not the complete suppression of localized corrosion but the way localized activity evolved and released electrochemical energy [36]. Figure 16 illustrates a schematic representation of the corrosion mechanisms proposed for the R and NP systems during the different stages of exposure.

During the first stage (3 to 35 days), both systems were predominantly governed by passive film stabilization. Although the NP system followed a similar passivation trend, it exhibited lower current amplitudes after the initial measurements. This behavior is consistent with the resistivity results obtained for the cement paste, where NP-modified specimens developed higher electrical resistivity than the R system. These findings suggest that passivation in the NP system occurred within a matrix that was inherently more resistive and less favorable to ionic transport [60,65].

The second stage (35 to 91 days) corresponded to the transition from passive stabilization to chloride-induced interfacial disturbance. This transition was particularly evident in the R system at 91 days, when the EN signals exhibited a pronounced transient, Rn reached a maximum, Zn displayed high impedance at low frequencies, and the HHT spectra revealed a localized high-energy contribution. Collectively, these observations indicate a critical electrochemical reorganization [70,71].

The third stage (91 to 154 days) provided the clearest differentiation between the corrosion mechanisms of the two systems. In the R system, shot noise parameters reached q magnitudes of the order of 10^−6^ C, indicating that localized corrosion events involving significant charge transfer prior to arrest or partial repassivation. In contrast, the NP system was dominated by lower-q events, primarily within the 10^−8^ to 10^−7^ C range, occurring at higher frequencies. These results suggest that the incorporation of NPs did not eliminate the localized corrosion but altered its electrochemical characteristics and reduced its intensity [33,73].

The wavelet and HHT results further support this interpretation. At 119 and 154 days, the NP system exhibited energy redistribution toward intermediate crystals, together with a broader distribution of energy across the time–frequency domain. This behavior is indicative of recurrent depassivation–repassivation processes, in which chloride-induced passive film disruption occurred locally, while the development of active anodic sites remained constrained by the higher resistivity and lower ionic connectivity of the NP-modified matrix.

By day 182, neither system had fully reestablished a passive state. Instead, both evolved toward a quasi-stable electrochemical regime characterized by a more organized and restricted response following a period of pronounced non-stationary activity. Although localized corrosion persisted, the NP system maintained a more constrained electrochemical behavior, reflecting the beneficial effect of nanoparticle incorporation in limiting ionic transport and mitigating the severity of localized corrosion processes.

## 4. Conclusions

This study investigated the corrosion behavior of steel embedded in concrete with and without SiO_2_ NP treatment using several methods of electrochemical noise analysis. The following conclusion can be drawn:

- The sol–gel synthesis route successfully transformed SCBA into NPs with a purity of 90.6%. The synthesized NPs were predominantly amorphous, exhibited an agglomerated nanometric morphology, and contained characteristic Si-O-Si functional groups.
- Electrical resistivity measurements and N_2_ adsorption analysis revealed that the NP system developed higher resistivity and altered pore accessibility, indicating a cementitious matrix with lower ionic connectivity.
- The reference (R) system exhibited a critical electrochemical transition at 91 days, whereas the NP system did not suppress localized corrosion but altered its severity, charge magnitude, and time–frequency characteristics.
- Shot noise analysis showed that the R system was dominated by higher-charge events, while the NP system exhibited lower-charge, more frequent transients.
- Wavelet and HHT analyses further demonstrated that corrosion-related energy in the NP system was dissipated more gradually and distributed over a broader range of time–frequency scales, particularly between 119 and 154 days. By day 182, neither system had fully regained passivity; instead, both evolved toward a quasi-stable electrochemical regime. Nevertheless, the NP system maintained a more restricted electrochemical response, indicating that nanoparticle incorporation mitigated the severity of localized corrosion by limiting ionic transport and reducing the intensity of depassivation events.

## Figures and Tables

**Figure 1:**
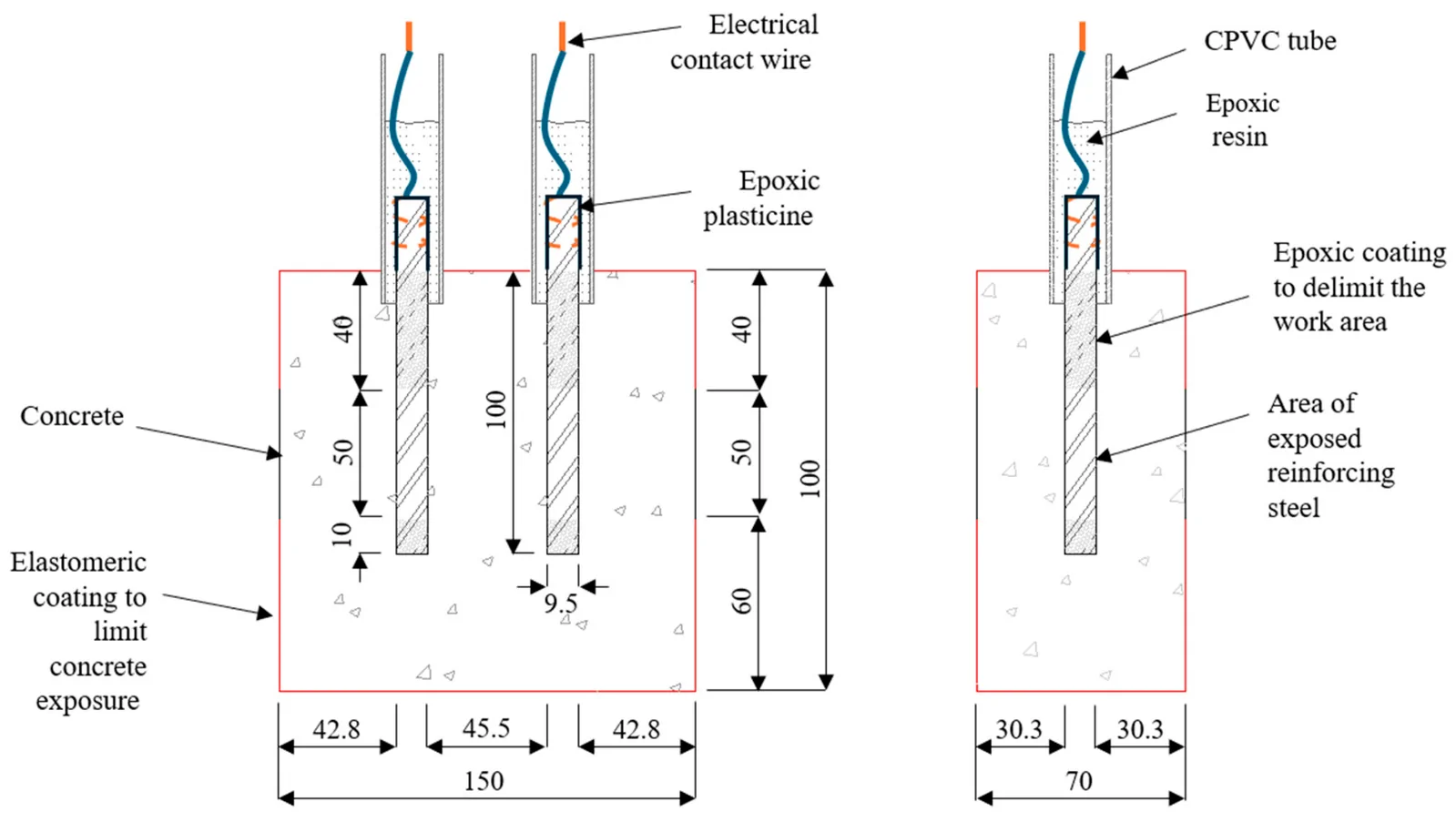
Schematic representation of the SC systems (dimensions in mm).

**Figure 2:**
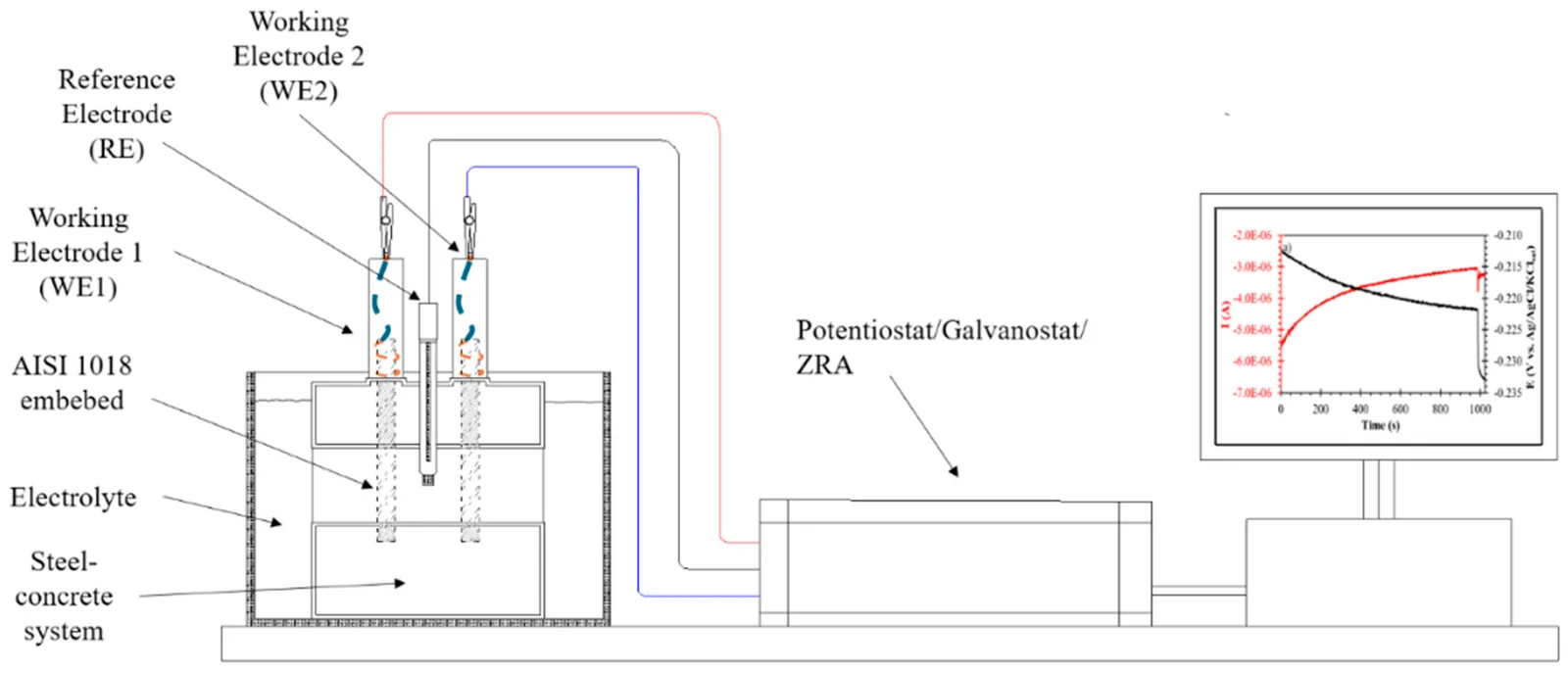
Experimental arrangement of EN measurements of SC systems.

**Figure 3:**
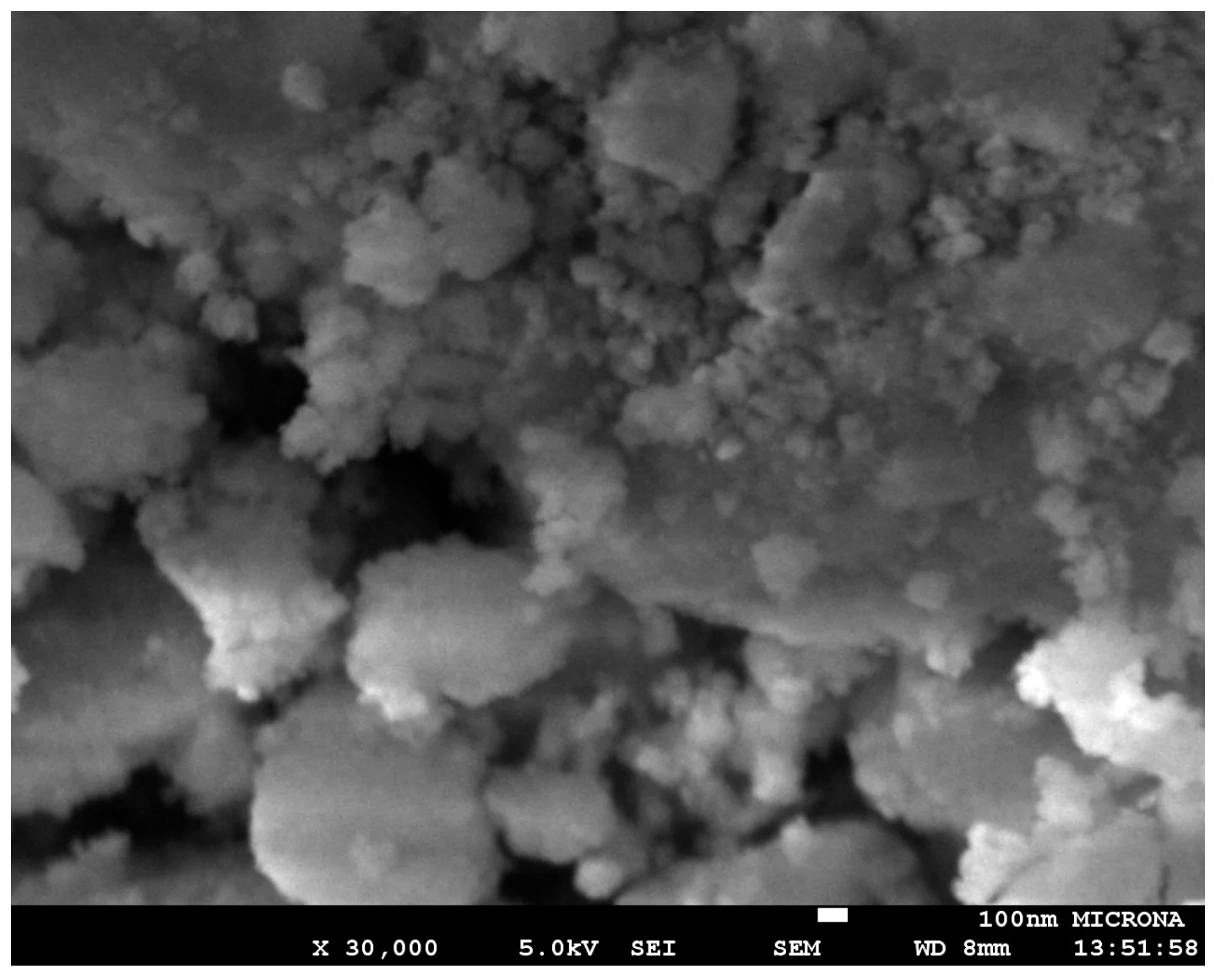
SEM micrograph of SiO_2_ NPs synthesized from SCBA.

**Figure 4:**
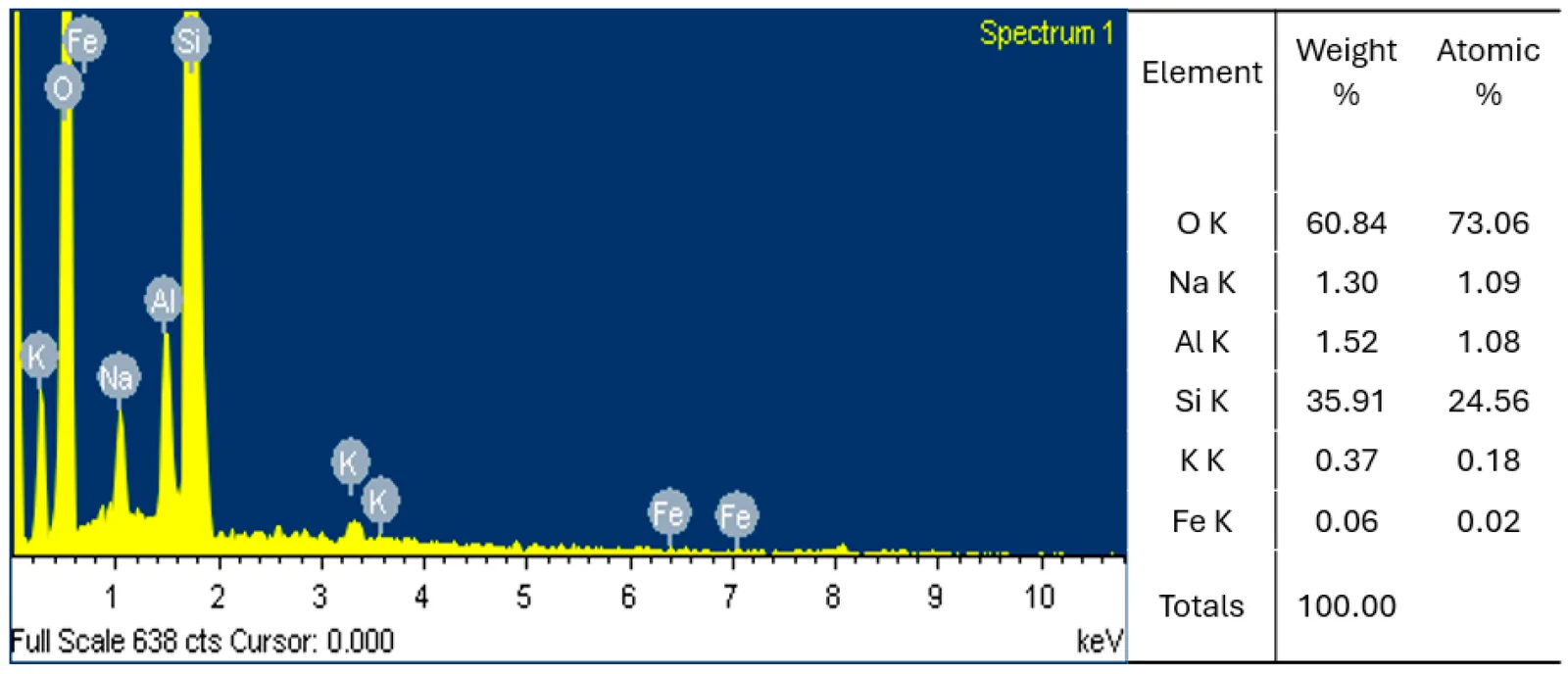
EDS of the SiO_2_ NPs obtained from SCBA.

**Figure 5:**
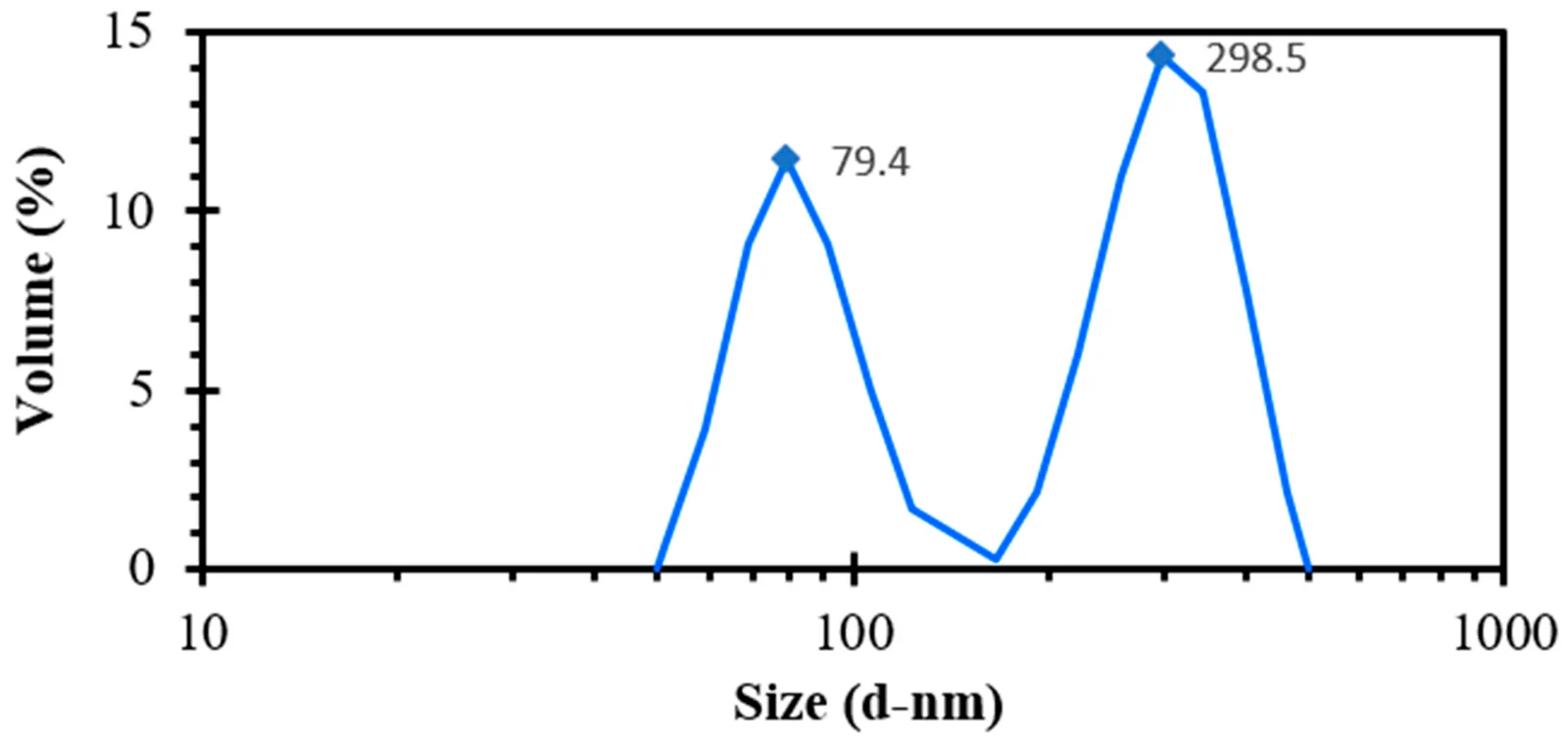
Size distribution by volume of SiO_2_ NPs.

**Figure 6:**
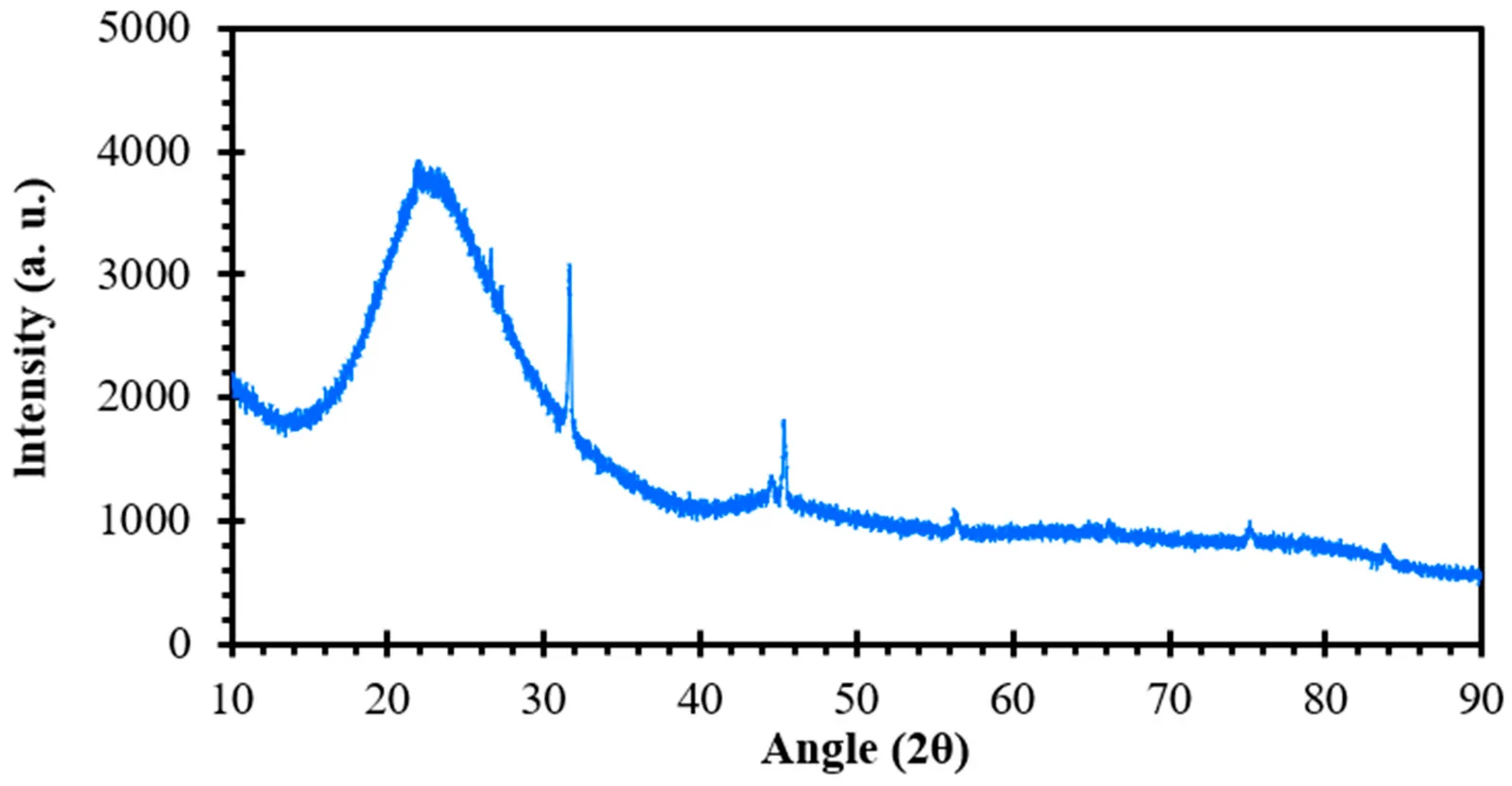
XRD diffractogram of SiO_2_ NPs.

**Figure 7:**
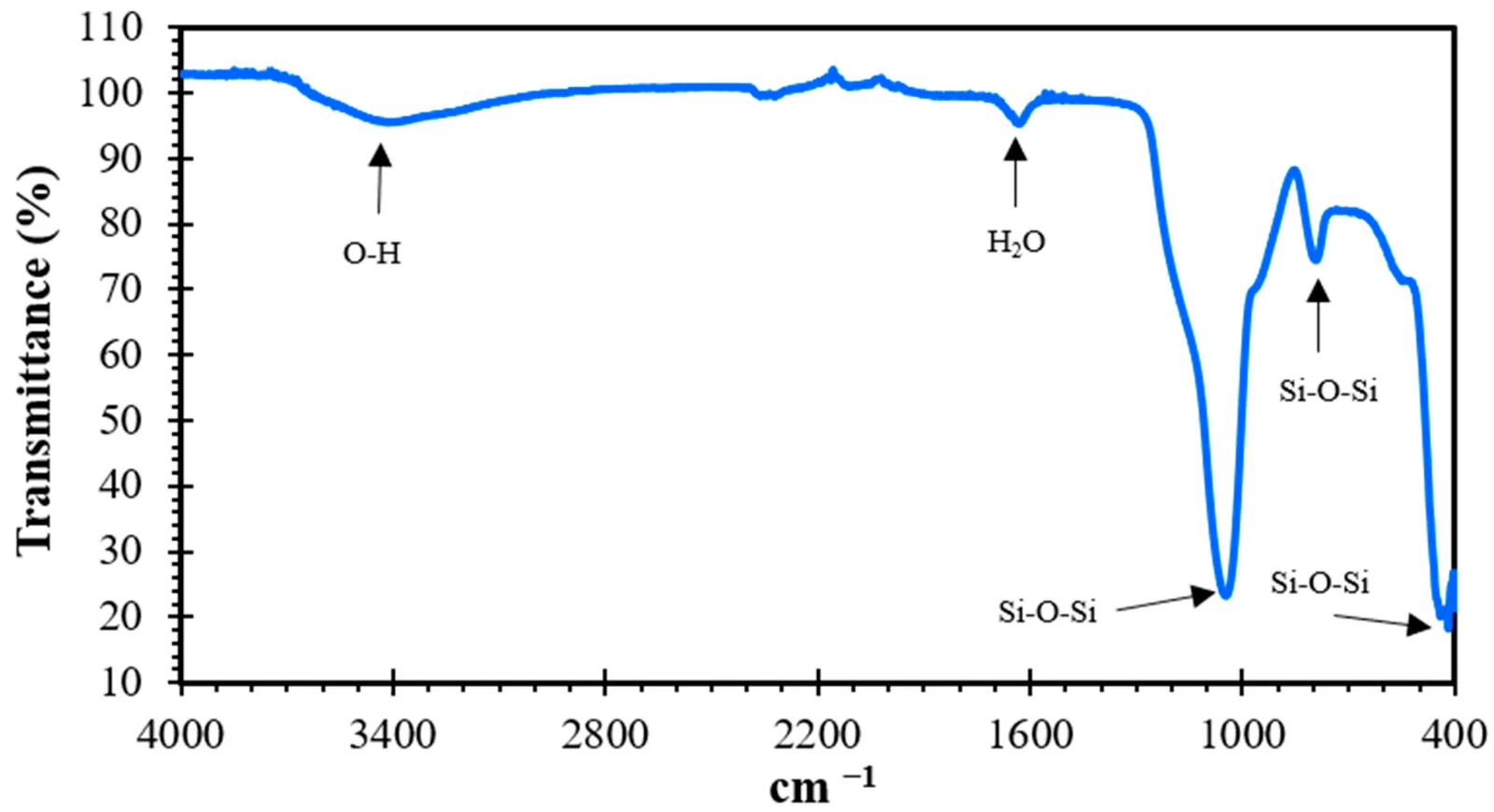
FTIR spectrum of NPs synthesized from SBCA.

**Figure 8:**
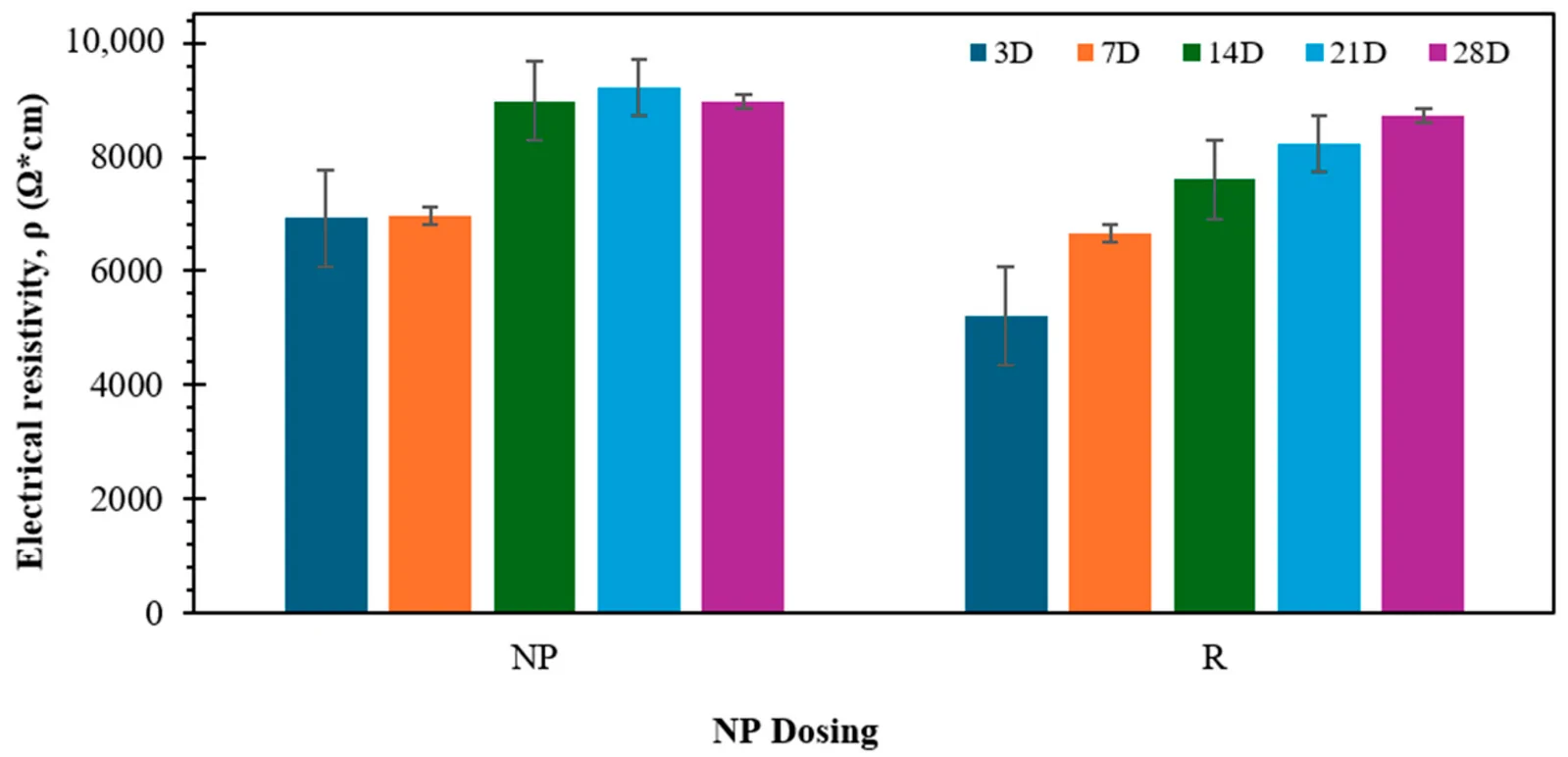
Electrical resistivity of R and NP paste.

**Figure 9:**
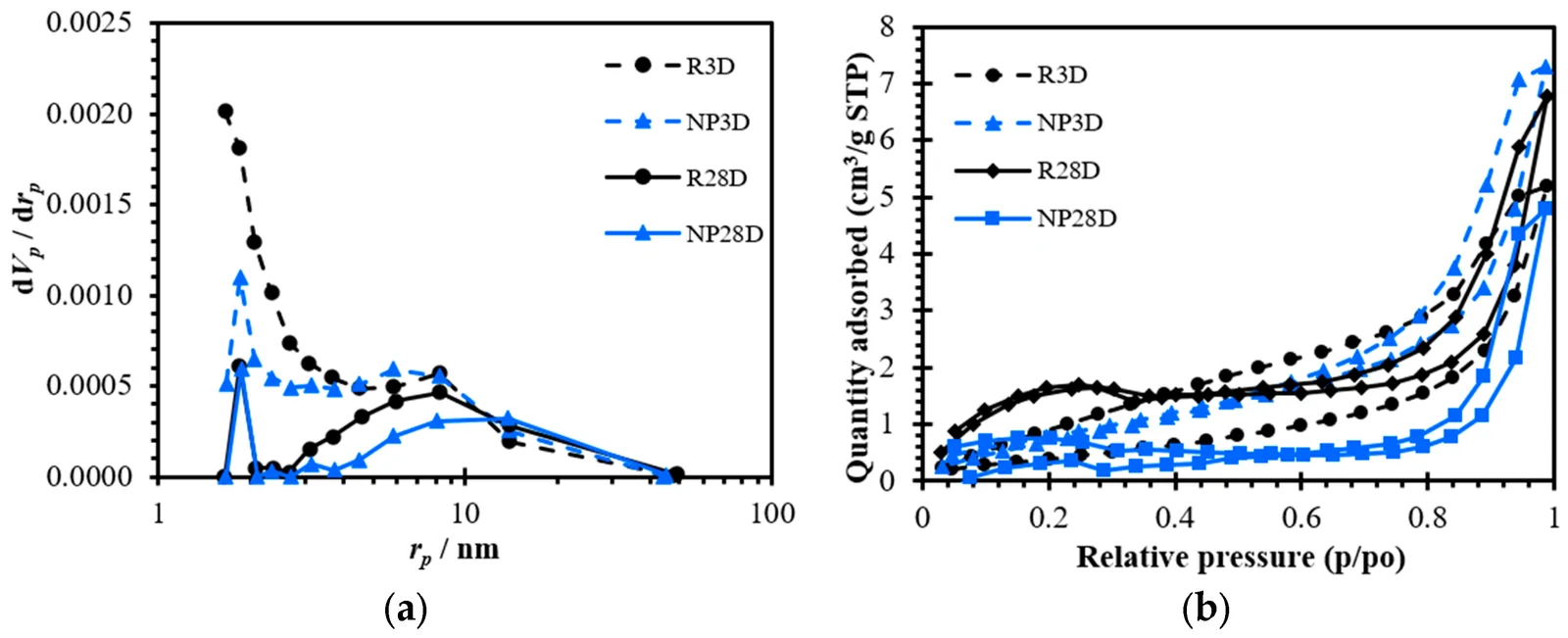
(**a**) Pore size distributions in R and NP paste and (**b**) adsorption isotherms of R and NP paste.

**Figure 11:**
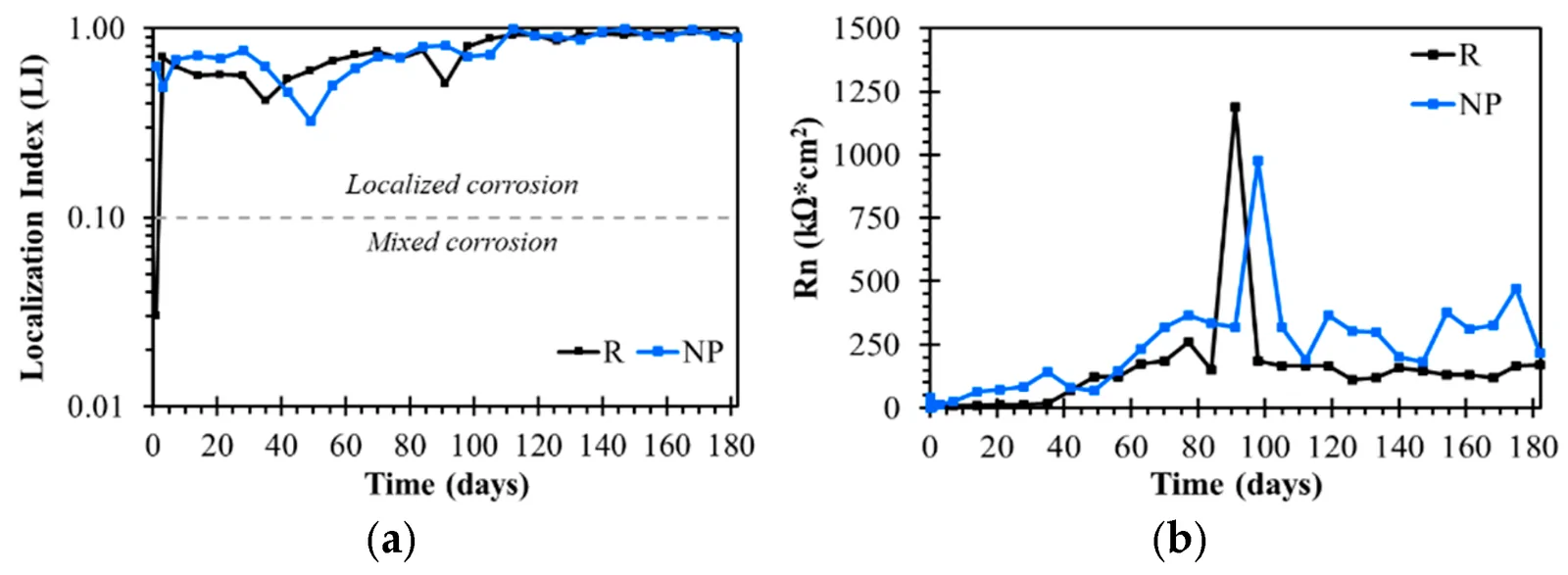
(**a**) Localization index and (**b**) noise resistance values for R and NP systems.

**Figure 12:**
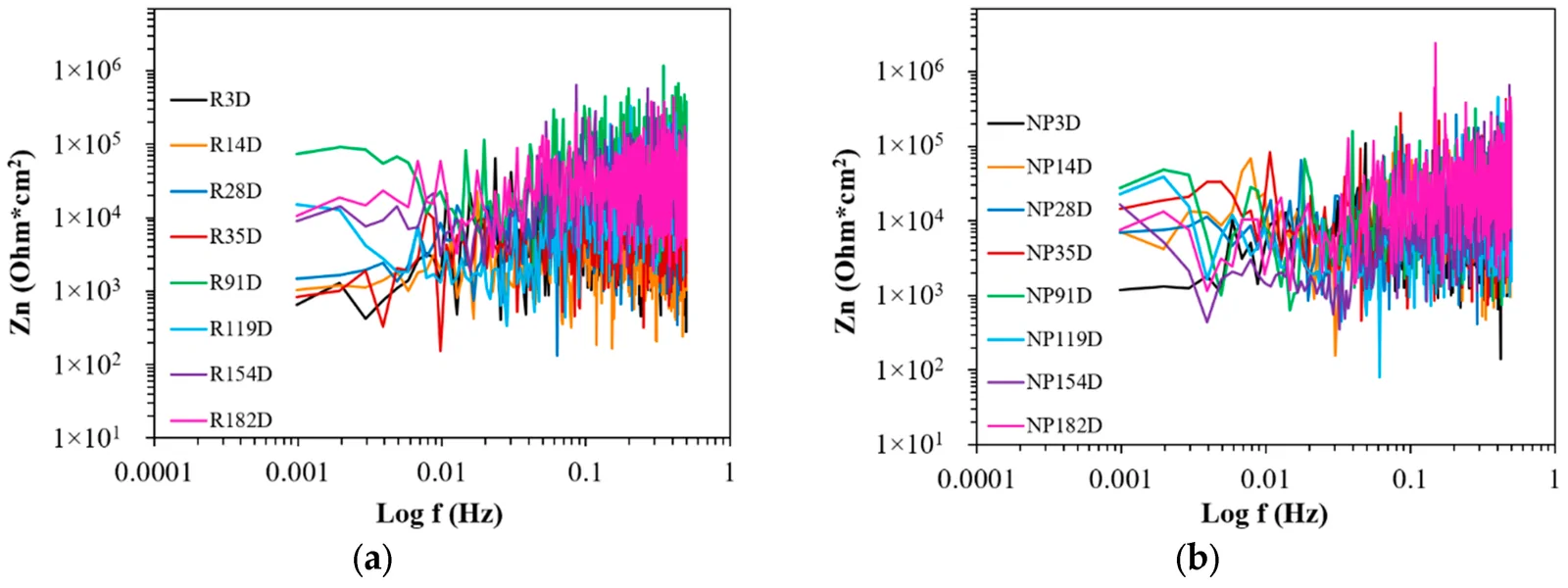
Noise impedance (Zn) values for (**a**) R and (**b**) NP systems.

**Figure 13:**
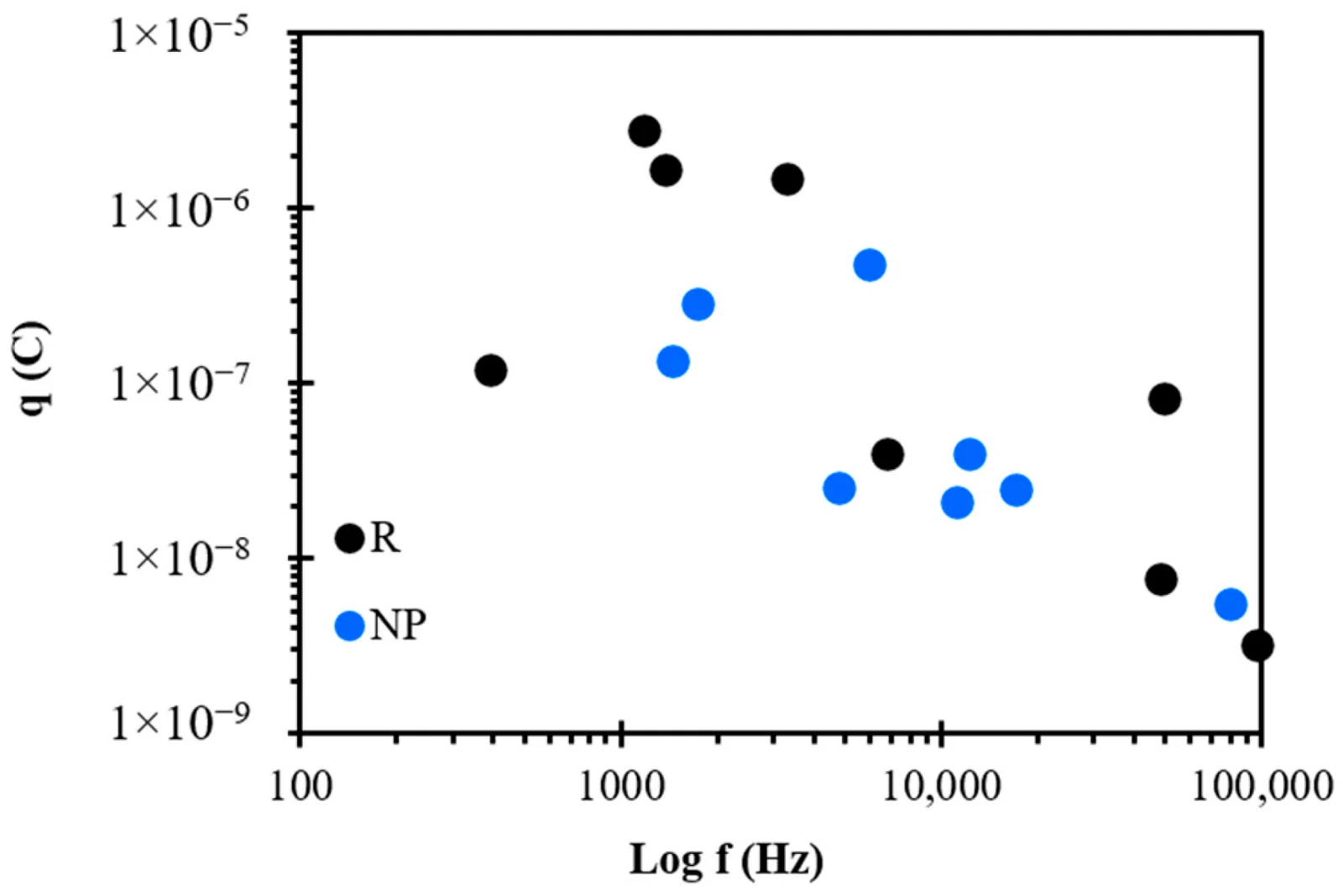
Shot noise parameters derived from EN signals for the R and NP systems.

**Figure 14:**
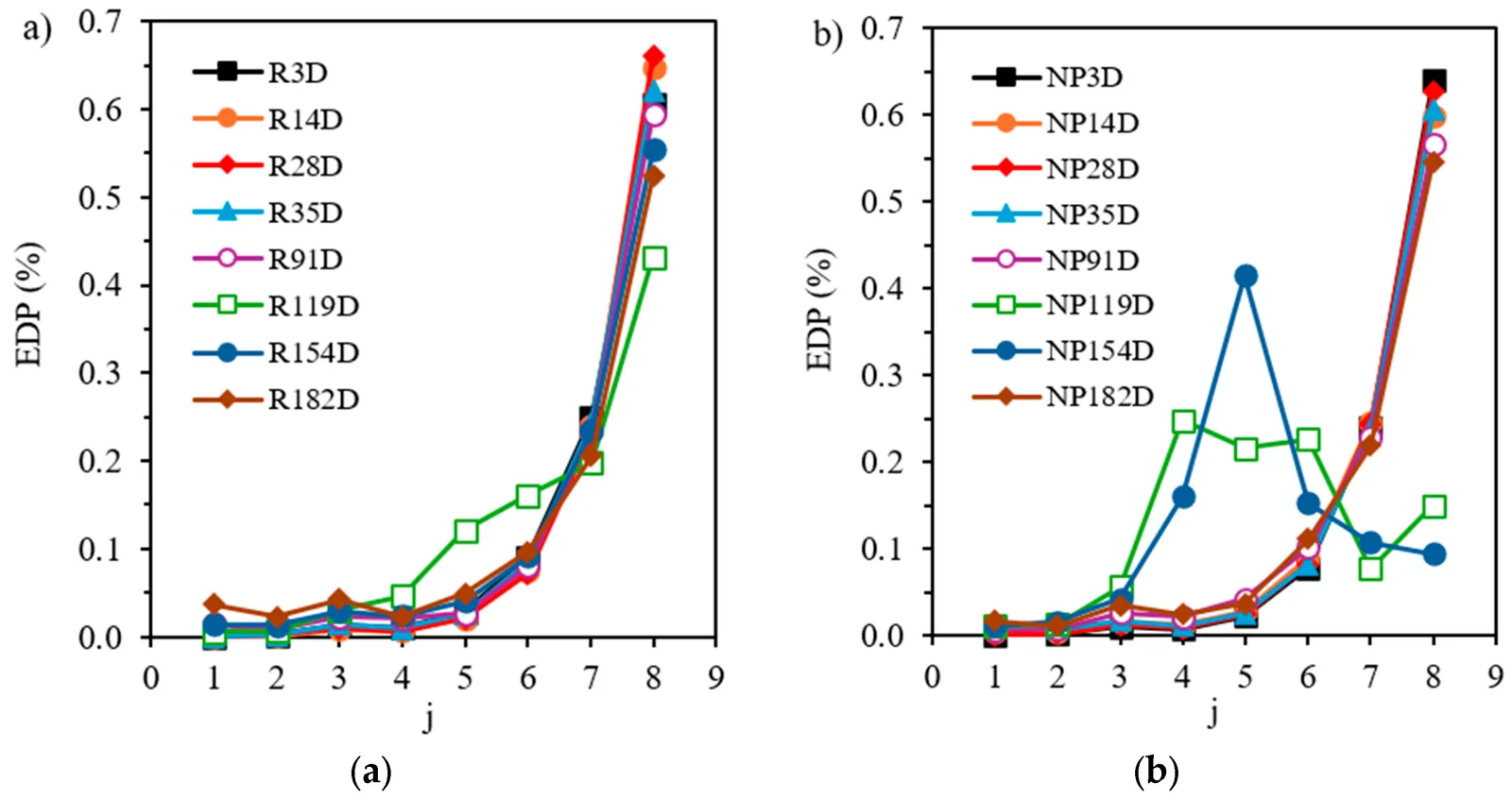
Energy distribution plots (EDPs) for the (**a**) R and (**b**) NP systems.

**Figure 15:**
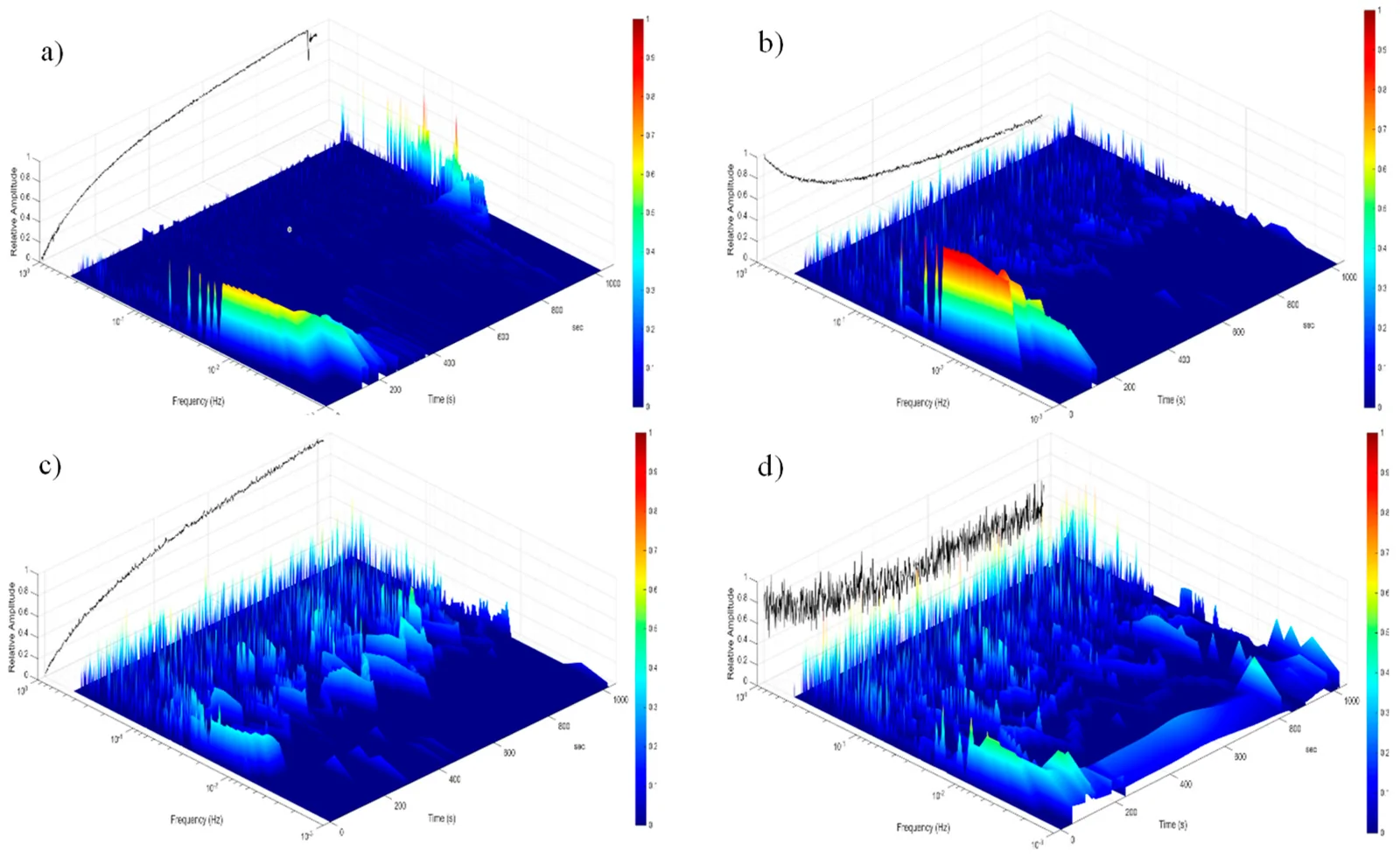

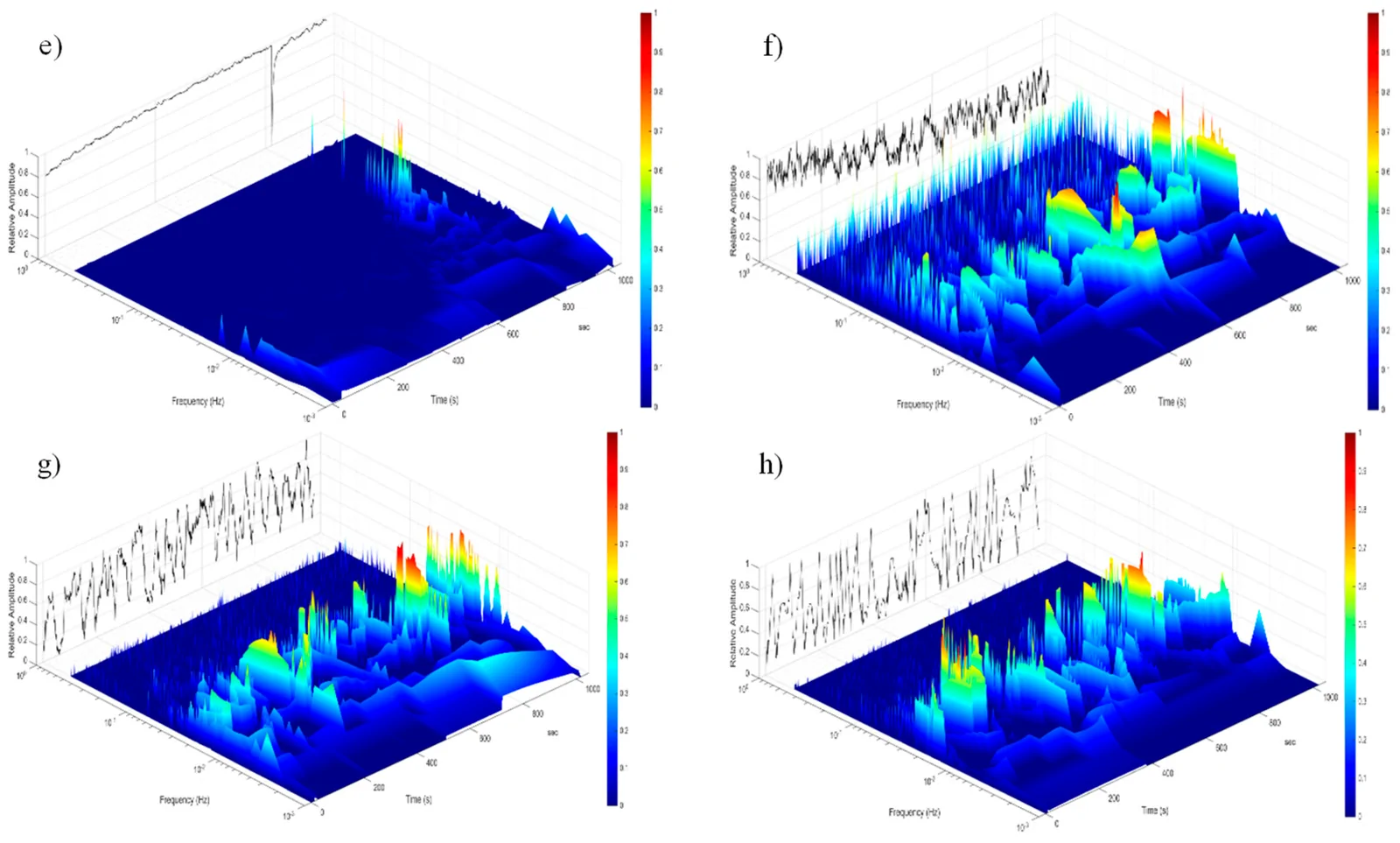
Hilbert–Huang transform for R (**a**,**c**,**e**,**g**,**i**) and NP (**b**,**d**,**f**,**h**,**j**) systems at different days of exposure: 3 (**a**,**b**), 35 (**c**,**d**), 91 (**e**,**f**), 119 (**g**,**h**) and 182 (**i**,**j**) days.

**Figure 16:**
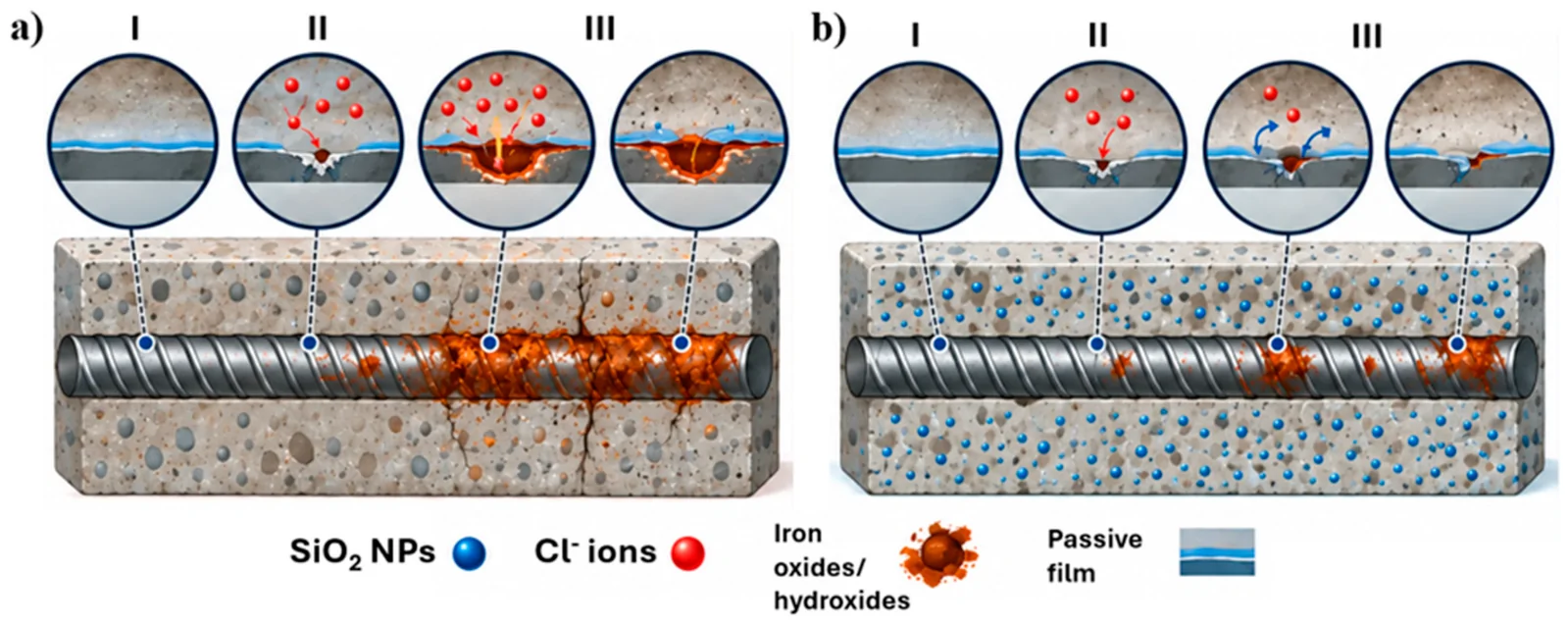
Schematic representation of the corrosion mechanism in the RCS: (**a**) R system and (**b**) NP system.

**Table 1:** Chemical composition of CPC 40.

Component	CaO	SiO_2_	Al_2_O_3_	Fe_2_O_3_	MgO	SO_3_	K_2_O	P_2_O_5_	TiO_2_	V_2_O_5_
**Concentration (%)**	72.35	13.75	3.47	3.79	0.23	4.60	1.02	0.52	0.25	0.03

**Table 2:** Concrete mix design.

Component	Cement	Water	Coarse Aggregate	Fine Aggregate	Superplasticizer	*w*/*c*
**Proportion** **(kg/m^3^)**	480	216	616	1037	4.5	0.45

**Table 3:** XRF of synthesized NPs.

Component	SiO_2_	CaO	Al_2_O_3_	Fe_2_O_3_	K_2_O	P_2_O_5_	Cl	TiO_2_
**Concentration (%)**	90.63	0.417	3.980	0.156	0.696	0.624	3.472	0.030

**Table 4:** Surface area and average pore diameter in R and NP paste.

Paste	BET Surface Area (m^2^/g)		Mean Pore Diameter (nm)		Pore Volume (cm^3^/g)	
	3D	28D	3D	28D	3D	28D
**R**	31.94	13.76	3.29	3.68	0.073	0.060
**NP**	23.90	9.83	3.69	3.72	0.069	0.048

## Data Availability

The data presented in this study are available on request from the corresponding author.
